# Proceedings of the 1st biannual bridging the gaps in lung cancer conference

**DOI:** 10.1093/oncolo/oyae228

**Published:** 2024-09-05

**Authors:** Narjust Florez, Sandip P Patel, Heather Wakelee, Lyudmila Bazhenova, Erminia Massarelli, Ravi Salgia, Brendon Stiles, Solange Peters, Jyoti Malhotra, Shirish M Gadgeel, Jorge J Nieva, Michelle Afkhami, Fred R Hirsch, Matthew Gubens, Tina Cascone, Benjamin Levy, Joshua Sabari, Hatim Husain, Patrick C Ma, Leah M Backhus, Puneeth Iyengar, Percy Lee, Russell Miller, Jacob Sands, Edward Kim

**Affiliations:** Dana-Farber Cancer Institute, Harvard University School of Medicine, Boston, MA, United States; Sanford Stem Cell Clinical Center and San Diego Center for Precision Immunotherapy, University of California San Diego, La Jolla, CA, United States; Stanford University School of Medicine and Stanford Cancer Institute, Stanford, CA, United States; University of California San Diego Moores Cancer Center, La Jolla, CA, United States; Department of Medical Oncology & Therapeutics Research, City of Hope, Duarte, CA, United States; Department of Medical Oncology & Therapeutics Research, City of Hope, Duarte, CA, United States; Cardiovascular and Vascular Surgery, Albert Einstein College of Medicine, Bronx, NY, United States; Medical Oncology, Lausanne University Hospital, Lausanne Vaud, Switzerland; Thoracic Medical Oncology, City of Hope Orange County, Irvine, CA, United States; Henry Ford Cancer Institute, Henry Ford Health Center, Detroit, MI, United States; Norris Comprehensive Cancer Center, Keck School of Medicine, University of Southern California, Los Angeles, CA, United States; Department of Pathology, City of Hope Comprehensive Cancer Center, Duarte, CA, United States; Icahn School of Medicine and Center for Thoracic Oncology, Mount Sinai Health System, New York, NY, United States; Thoracic Medical Oncology, University of California San Francisco, San Francisco, CA, , United States; University of Texas MD Anderson Cancer Center, United States; Sidney Kimmel Comprehensive Cancer Center, Johns Hopkins University School of Medicine, Baltimore MD, United States; Langone Health Perlmutter Cancer Center and NYU Langone Grossman School of Medicine, New York, NY, United States; University of California San Diego, San Diego, CA, United States; Penn State Cancer Institute, Milton S. Hershey Medical Center and Penn State College of Medicine, Hershey, PA, United States; Department of Cardiothoracic Surgery, Standford University, Palo Alto, CA, United States; Memorial Sloan Kettering Cancer Center, New York, NY, United States; City of Hope National Medical Center, Los Angeles, CA, United States; University of California San Diego, San Diego, CA, United States; Dana-Farber Cancer Institute, Harvard University School of Medicine, Boston, MA, United States; Department of Medical Oncology & Therapeutics Research, City of Hope, Duarte, CA, United States

**Keywords:** clinical decision-making, controversies, unanswered questions, lung cancer diagnosis, lung cancer treatment, lung cancer management

## Abstract

Lung cancer is the leading cause of cancer death in the US and globally. The mortality from lung cancer has been declining, due to a reduction in incidence and advances in treatment. Although recent success in developing targeted and immunotherapies for lung cancer has benefitted patients, it has also expanded the complexity of potential treatment options for health care providers. To aid in reducing such complexity, experts in oncology convened a conference *(Bridging the Gaps in Lung Cancer*) to identify current knowledge gaps and controversies in the diagnosis, treatment, and outcomes of various lung cancer scenarios, as described here. Such scenarios relate to biomarkers and testing in lung cancer, small cell lung cancer, *EGFR* mutations and targeted therapy in non-small cell lung cancer (NSCLC), early-stage NSCLC, *KRAS/BRAF/MET* and other genomic alterations in NSCLC, and immunotherapy in advanced NSCLC.

## Teaser

The development of targeted and immunotherapies for lung cancer has introduced treatment complexities for health care providers. This article describes the knowledge gaps and controversies in the diagnosis, treatment, and outcomes of various lung cancer scenarios, as identified and discussed by prominent experts in oncology at the recent conference “Bridging the Gaps in Lung Cancer.”

**Implications for practice**

Though contemporary success in developing targeted and immunotherapies for lung cancer has benefitted patients, it has also increased the complexity of the treatment landscape for health care providers. Considering the array of possible oncogenic drivers of non-small cell lung cancer (, proteomic abnormalities, disease stage, treatment history, and biomarkers, oncology teams may face challenges in selecting potential treatment combinations. To reduce such challenges, we describe the knowledge gaps and controversies in the diagnosis, treatment, and outcomes of various lung cancer scenarios, as identified and discussed by prominent experts in oncology.

## Preface

Lung cancer is the leading cause of cancer death and the leading cause of death before age 70 in the US.^1^ Mortality from lung cancer has been declining, attributed to a reduction in incidence and advances in treatment.^1,2^ Treatment advances of the most common histological subtype of lung cancer, non-small cell lung cancer (NSCLC), have accelerated the decrease of mortality, compared to the rate of decreasing incidence, through novel-targeted therapies and immunotherapies.^3-5^ Small cell lung cancer (SCLC) has had fewer treatment advances, and by contrast, the recent decline in mortality rates are largely related to decline in incidence.^5^ These effects are supplemented by the reduction of smoking rates over the same periods.^5^

Although recent success in developing novel treatments for lung cancer has benefitted patients, it has also increased the complexity of the treatment landscape. As of 2023, there were dozens of therapies approved for NSCLC against more than 10 key molecular pathways. SCLC approvals have also accelerated in the past decade as understanding of the disease and immunotherapies has continued to evolve.^6,7^ The growing number of neoadjuvant and EGFR-directed therapies have further expanded the potential treatment options available.

## Methods and Objectives

The 2023 inaugural *Bridging the Gaps in Lung Cancer* conference gathered a panel of oncology experts as a working group (WG) to identify prevailing challenges, knowledge gaps, and controversies in the field of lung cancer. The WG included multidisciplinary oncology experts from academic cancer centers and community practices across geographic regions of the US. Specialists from thoracic surgery, radiation oncology, translational medicine, interventional pulmonology, and medical oncology were represented, enabling a comprehensive exploration of issues surrounding lung cancer diagnosis and treatment.

The conference adopted a focused, single-stream meeting format to provide a forum for examination of areas of lung cancer management where there is limited or no consensus in clinical practice. There were 7 sessions on a range of lung cancer topics selected in advance by W.G. faculty members, including co-chairs (E.K. and N.F.) and session chairs (S.P., H.A.W., L.B., E.M., R.S.), during a series of virtual pre-conference workshops. Faculty speakers delivered independently developed presentations as outlined in Table 1, then an open panel discussion, moderated by the co-chairs and session chairs, was convened at the end of each session to facilitate conversation on critical knowledge gaps and controversies that emerged. This structure provided a platform for examination of presented data, shared experiences, and divergent viewpoints.

**Table 1.** Meeting presentation topics.

1. Biomarkers and testing in NSCLC and SCLC	Program Chairs: E. Kim, N. Florez
What, when, and how to test	
2. SCLC	Session Chair: E. Maserelli
Immunotherapy in SCLC Emerging treatment options in SCLC	
3. *EGFR* mutations and targeted therapy in NSCLC	Session Chair: L. Bazehnova
*EGFR* targeting therapies in advanced NSCLC Uncommon *EGFR* mutations in NSCLC and their treatment Treatment strategies for patients progressing on EGFR TKIs (continuing and moving to combination strategies)	
4. Early NSCLC	Session Chair: H. Wakelee
Which patients with NSCLC are resectable? Neoadjuvant therapy in NSCLC Adjuvant immunotherapy and targeted therapy Treatment of stage IIIB NSCLC	
5. Targeting KRAS/BRAF/MET mutations and fusions in NSCLC	Session Chair: R. Salgia
KRAS mutations in NSCLC and their treatment BRAF mutations in NSCLC and their treatment MET skipping mutations in NSCLC and their treatment ALK and ROS1 rearrangements in NSCLC and their treatment NTRK and RET fusions in NSCLC and their treatment	
6. Immunotherapy in advanced NSCLC	Session Chair: S. Patel
Chemo-immunotherapy in advanced NSCLC Dual IO inhibition in the treatment of NSCLC Treatment options for patients who progress on immunotherapy Emerging therapies and emerging controversies in advanced NSCLC	
7. Closing discussion and summary	

Abbreviations: 1L, first line; 2L, second line; ADC, antibody-drug conjugate; ALK, anaplastic lymphoma kinase; BRAF, v-raf murine sarcoma viral oncogene homolog B1; CNS, central nervous system; EGFR, epidermal growth factor receptor; IO, immune-oncology; KRAS, Kirsten rat sarcoma viral oncogene homologue; MET, proto-oncogene for a receptor tyrosine kinase; MRD, minimal residual disease; NSCLC, non-small cell lung cancer; NTRK, neurotrophic tyrosine receptor kinase; PD-L1, programmed death ligand 1; RET, rearranged during transfection receptor kinase; ROS1, receptor tyrosine kinase encoded by the c-ros oncogene; RWE, real-world evidence; SCLC, small cell lung cancer; SWOG, Southwest Oncology Group; TKI, tyrosine kinase inhibitor.

This *Proceedings* report represents the collective discussion of meeting participants on the knowledge gaps and controversies in evidence and clinical practice. The insights presented here will inform a follow-up conference in 2024 to develop formal consensus recommendations using a modified Delphi approach to help bridge clinical gaps.^8^

### Biomarkers and molecular testing

Predominant areas of unmet need are outlined in Table 2

**Table 2.** Key gaps and needs in biomarkers and molecular testing.

a. Characterization and validation (relevance/predictive) b. Guidelines or standardization for testing reports and interpretation c. Application for never smoker vs. prior history of smoking d. Classification of disease subtypes

Robust biomarkers may allow for minimally invasive detection of cancer, risk stratification of patients based on molecular profiles, and provide pathological information around indeterminate nodules or tumors assessed by low-dose computed tomography screening.^9,10^ However, gaps were identified that must be addressed before their full value can be recognized.

Better characterization of biomarker expression patterns and testing platforms is required to appropriately inform treatment decisions and risk stratification. Validation of the predictive value of existing biomarkers is needed and could be supported by standardized test methods. Both biomarker characterization and validation could be facilitated by prospective clinical and real-world (RW) studies, as well as more big data efforts to identify molecular or histopathologic signatures that can inform development of cross-platform assays and artificial intelligence models.

Molecular testing is underutilized despite technological advances. The panel identified challenges with testing, including tissue acquisition and processing; limited time for testing, treatment delays due to aggressive disease, and insurance coverage gaps. Despite more than 15 years of development, the specialized next generation sequencing (NGS) equipment, platforms, and technical expertise are still not fully accessible across institutions, and clinical application is not standardized.^11^ There was a lack of agreement on benefits and use of sequential screening. While panelists agreed NGS should be the minimum diagnostic testing standard beyond lung scans, time is a key consideration—regardless of the collection method, NGS analysis and testing generally delays treatment by weeks. Clinical standards for NGS and biomarker testing could reduce turnaround times.

Standardization of testing reports, report analysis, and guidelines for translating testing results are also needed to inform clinical decision-making. There are limited guidelines specifying techniques, sequencing, or analysis for molecular tests, resulting in highly variable practice.^11^ Exploration of molecular testing in the context of smoking status is necessary to identify potential genetic links to disease or treatment response.

The high mitotic rate in SCLC tumors drives heterogeneity and acquired resistance, making it difficult to identify targetable biomarkers.^7,12^ Limited tissue availability for SCLC is a challenge in all aspects of biomarker development and testing. Liquid biopsy offers hope to leverage the high circulating tumor DNA (ctDNA) often found in SCLC, although more clinical validation of associated prognostic signatures is required.^13^ Further, there is general agreement on SCLC subtypes, but their treatment implications remain unclear. Subtype molecular testing methods require further refinement, particularly for the inflamed (SCLC-I) subtype.^14,15^

## Small cell lung cancer

Predominant areas of unmet need are outlined in Table 3

**Table 3.** Key gaps and needs in small cell lung cancer.

1. Pragmatic clinical trial design Leveraging cancer research networks like SWOG Inclusion of RW patient populations, biomarker testing endpoints, differential response assessments (eg, brain vs full body) 2. Clinical practice guidance for treatment selection and patient management Selecting therapies based on deeper understanding of the underlying biology Clarifying parameters for maintenance therapy (and adding therapies) Education community oncologists on new agents and appropriate use/toxicity management as new treatments transition from academic centers Managing patients with transformed SCLC—identifying preferred therapeutic agents

Abbreviations: RW, real-world; SCLC, small cell lung cancer; SWOG, Southwest Oncology Group.

### Clinical trial design

There is a growing need for more pragmatic clinical trial design in SCLC. Current randomized clinical trials (RCTs) are primarily designed to answer one question at a time, resulting in slow data accumulation. Conducting trials through a cancer research network such as SWOG, Southwest Oncology Group (SWOG), like the ADRIATIC study, can help maximize impact and expand reach.^16,17^ Converging studies into a translational “umbrella” approach could also expedite the process. A biomarker-focused study would be helpful to explore multiple characterization and validation questions simultaneously, but funding could be challenging. SWOG S1929 was completed as a biomarker-driven trial in the first-line setting, incorporating a PARP inhibitor in the setting of high SLFN11 expression.18 Although the study did not meet efficacy endpoints, it serves as a feasibility demonstration of a SCLC biomarker-driven trial.

The panel noted that improvements in patient selection are required, as RCT patient populations often do not properly reflect the RW population receiving the treatment, and women and minority populations are commonly underrepresented. Further, eligibility criteria and endpoints that inform treatment sequencing, dose modifications, concurrent therapy use, or measurable residual disease (MRD) correlates, are necessary to accelerate changes in practice. This is particularly important for patients with limited-stage disease. Novel trial design suggestions included investigating subpopulations most suitable for immunotherapy or distinguishing brain-only from full-body progression and response.

### Clinical practice: treatment selection and patient management

Progress in SCLC precision medicine has been historically slow; chemotherapy was the mainstay of 1L and 2L treatment for decades until only recently, as understanding of the molecular mechanisms of disease started to evolve.^18^ There are now a variety of therapeutic modalities approved and in development. The targeted nature of many of these therapies offers promise for improved outcomes, but the varied mechanisms of action make treatment selection and patient management increasingly complex.

Treatment selection and sequencing based on disease biology could benefit from consensus and guidance. The 2L + setting is particularly challenging. Recent advances, such as inclusion of programmed death ligand 1 (PD-L1) inhibitors with platinum and etoposide in the treatment of SCLC, have shown an increase in durable responses to 1L treatment.^19,20^ However, many patients progress and have poor outcomes on maintenance immunotherapy, so clarifying parameters and strategies are needed.^21^ Although topotecan is the standard 2L therapy, panelists debated optimal agents and combinations for 2L + given the similar cytotoxicity of available options. Education on toxicity management and appropriate use is needed for new treatments as they transition from academic centers. Lurbinectedin was noted as a potential advance in 2L treatment with improved efficacy and safety that is currently being explored through trials and combination strategies.^22^ Additionally, management of patients with transformed SCLC can be a challenge, and it was noted that taxanes work well.

## *EGFR*-mutated NSCLC

Predominant areas of unmet need are outlined in Table 4

**Table 4.** Key gaps and needs in *EGFR*-mutated non-small-cell lung cancer.

1. Guidance for frontline therapy selection 2. Treatment strategies upon progression for classical *EGFR* mutations a. Standards for managing treatment selection post-progression if: − An acquired abnormality is identified (*MET/RET/BRAF,* EGFR 797S) − No acquired abnormality is identified (TKI + chemotherapy in patients with versus without brain metastases; switch to chemotherapy and return to TKI after 4 cycles, role of amivantamab/chemotherapy?) Establish the role of 3rd generation TKI + amivantamab Establish the role of immunotherapy Guidance for oligoprogression Coordinated care with neurology and radiation 3. For uncommon *EGFR* mutations and resistance: Awareness and screening for uncommon alterations Better validation of uncommon *EGFR* mutations, understanding of clinical implications, and standardization of reporting Further investigations of resistance mechanisms Generation of more survival and RW data 4. Monitoring Guidance for CNS monitoring frequency (with or without brain metastases) More data on the utility of ctDNA

Abbreviations: CNS, central nervous system; RW, real-world; SCLC, small cell lung cancer; SWOG, Southwest Oncology Group; TKI, tyrosine kinase inhibitor.

### Frontline therapy

With evidence showing that checkpoint inhibitors have limited benefit for patients with *EGFR*-mutated (EGFRm) NSCLC, it remains unclear whether to initiate immunotherapy while waiting for screening.^23^ However, a minority of patients see sustained benefit with immunotherapy, so it is critical that clinical studies explore a role for these agents in the context of specific subpopulations, such as those with high *PD-L1* expression or tumor mutational burden (TMB), and as part of treatment combinations.^24^ It was also noted that frontline radiation must be considered during study design, and biology of disease should be considered for optimal therapy selection and timing.

### Treatment strategies upon progression

For patients with classical *EGFR* mutations and progressive disease, a treatment algorithm is needed for specific populations that do not have well-defined management strategies, such as when an acquired genetic abnormality is identified (eg, *MET/RET/BRAF*, *EGFR* 797S) or when patients have brain metastases. Further, the role and appropriate sequencing or combinations of third-generation tyrosine kinase inhibitors (TKIs), amivantamab, and chemotherapy must be better delineated, particularly with the recent approval of amivantamab + chemotherapy as 2L therapy. ADCs may also need to be considered for treatment algorithms with the conditional approvals of patritumab deruxtecan and telisotuzumab-vedotin.^25^

For patients with oligoprogression, which is generally regarded as ≤ 5 radiologically progressive lesions with otherwise stable poly- or oligometastatic disease, enhanced biologic definition of disease and guidance on treatment with resection versus systemic therapy is warranted.^26^ Coordinated care, involving specialists from neuro-oncology and proton centers, was suggested to inform treatment.

### Uncommon mutations and resistance

As genetic heterogeneity within the EGFRm subtype of NSCLC exists, it is critical to be aware of, and screen for, other genetic alterations besides the classical *EGFR* (exon 19) mutation. Most uncommon mutations are between exons 18 to 21, and exon 20 insertions specifically represent 4%-12% of the *EGFR* alterations seen in *EGFR*-driven NSCLC.^27^ Patients with exon 20 insertions have limited benefit from standard EGFRm TKIs or immunotherapy and have more promising options with the FDA approvals of amivantamab and mobocertinib as 2L therapy.^24,27^ Better validation of uncommon *EGFR* mutations, understanding of clinical implications, and standardization of reporting is needed to aid treatment decisions.

Resistance is another challenge with treatment of *EGFR*m NSCLC; resistance can arise from acquisition of new genetic mutations, epigenetic alterations, or nongenetic mechanisms, all of which require further investigation. More survival and RW data are also needed to inform clinical decision making around uncommon mutations. The UNICORN study is a model example that explored RW survival and resistance in the context of *EGFR* mutations, showing that compound EGFRm NSCLC (containing both uncommon and common *EGFR* mutations) had better outcomes with the third-generation TKI osmertinib.^28^

### Monitoring

The incidence of central nervous system (CNS) metastases in patients with NSCLC is increasing, possibly due to therapies that are prolonging survival and increased detection through neuroimaging advancements.^29^ This incidence appears comparable between the EGFRm and wild-type NSCLC populations, but associations between EGFRm status, CNS metastases, and outcomes continue to be explored.^29-32^ Monitoring for CNS metastases requires standardization; some panelists use a 6-month frequency, but an evidence-based cadence is needed for patients with and without CNS metastases that is covered by insurance. Incorporating intracranial response criteria and patients with CNS metastases into prospective clinical trials can help bridge this evidence gap.^33^ Additionally, more data are needed on how to best utilize circulating tumor DNA (ctDNA), (using oncogenic driver mutations to monitor TMB, tumor-informed multiplex panels, etc.) as part of patient monitoring.

## Early-stage NSCLC

The predominant areas of unmet need in early-stage NSCLC are outlined in Table 5

**Table 5.** Key gaps and needs in early-stage non-small-cell lung cancer.

1. Patient identification and treatment strategies • Increase utilization of lung cancer screening; advise payers to include screening in wellness programs − Research in the utilization and benefits of lung cancer screening in light- and non-smokers • Standardize staging and use of methods: endobronchial ultrasound; mediastinoscopy; interventional pulmonology • Guidance or standards for primary therapy selection and outcome evaluation • Determine if chemotherapy/immunotherapy is universally needed, or if immunotherapy alone (and which) will suffice in some patients • Guidance on de-escalation 2. Perioperative strategies • Driver mutation characterization in clinical trials to inform utility in clinical decision making − Guidance on who should/shouldn’t receive targeted therapy and/or immunotherapy pre/post primary therapy − Guidance on which molecular targets will be challenging or require a different approach • Standardization of *PD-L1* staining • Hone optimal treatment; reduce risk of overtreatment; identify patients for de-escalation (timing/method) − Determine appropriateness of surgery, radiation, and systemic treatment first • Give guidance on which molecular targets will be challenging and require a different approach • Develop consensus around treatment of multifocal disease with adjuvant approaches. • Which IO monotherapy maximizes overall survival in cases of high *PD-L1* expression? • Post-hoc, meta-analyses issues: − Equal treatment of patients with complete pathological response vs. maximum pathological response? Perioperative strategy? • Registry database/RWE studies: − Validation of MRD measures with other diagnostic tests − Determine the perioperative clinical decision-making value of MRD measures • Refine the use of perioperative therapy in patients without a smoking history 3. Coordinated care • Full integration of cardiothoracic surgeons, thoracic surgeons, radiation oncologists, pulmonologists, pathologists, and rest of care team in developing criteria/standards of care • Multidisciplinary conferences that include all above disciplines • Delineated roles and responsibilities across specialties and reduced treatment delays through utilization of multidisciplinary tumor boards or, at a minimum, multidisciplinary discussions − Include representatives from community cancer centers and academic institutions that support case referrals − Referral of patients to academic centers when indicated • Opportunities for patient-based consultation between colleagues • Cross-institution committees and quality measures

Abbreviations: IO, immune-oncology; MRD, minimal residual disease; PD-L1, programmed death ligand 1; RWE, real-world evidence.

### Patient identification

One challenge in treating early-stage NSCLC is early diagnosis. Lack of education about screening guidelines, lack of insurance coverage, electronic medical record failures, time constraints, and negative perceptions on behalf of patients or providers, are common barriers that must be addressed.^34^ Advising insurers to include screening in wellness programs, researching benefits of screening for light and non-smokers, and standardizing screening and staging techniques may increase utilization.

In initial stages of NSCLC, defining a set of guidelines across institutions to identify candidates for resection would help standardize decisions regarding primary therapy and evaluate outcomes between patient populations. Characterization of driver mutations and additional data from RCTs and RW studies are essential to better inform treatment selection, such as if combination chemotherapy/immunotherapy should be used broadly, or if immunotherapy alone will suffice for some patients. Further, identifying the appropriate timing and methods for de-escalation is needed.

### Perioperative strategies

Perioperative strategies, such as neoadjuvant and adjuvant therapy, have shown promising results and are being investigated in early-stage NSCLC trials like ADAURA-2.^35,36^ Guidance is lacking around the use of neoadjuvant only, adjuvant only, or a combination approach, and where immunotherapy is best placed in a perioperative strategy. In that context, it is unclear how *PD-L1* expression and driver mutations other than *EGFR* and *anaplastic lymphoma kinase* (*ALK*) play into decision making. Recent studies suggest there is relative consistency in *PD-L1* staining across institutions using common assays,^37^ so there may be an opportunity to standardize. Regarding *PD-L1* staining, the PACIFIC-4 trial (NCT03833154) and SWOG (see https://www.swog.org) will generate a much-needed body of evidence in outcomes of neoadjuvant versus adjuvant therapy in the context of *PD-L1* expression and may guide its interpretation in those different settings. Other unanswered questions include: Who needs additional adjuvant immunotherapy after neoadjuvant immunotherapy and how does *PD-L1* and pathologic complete response play into that decision process? What is the optimal duration of targeted therapy in those with resected driver *EGFR + *or *ALK + *NSCLC in the adjuvant setting?

Other areas in need of exploration related to perioperative strategies include the potential role of adjuvant chemotherapy, the value of MRD for clinical decision making, and the use of neoadjuvant therapy in non-smokers. The panel noted that despite some neoadjuvant trials showing MRD + recurrence, various testing platforms were used across trials, so a standard diagnostic test or confirmation with orthogonal methods is warranted. Pooling of data across trials, platforms, or tissue repositories was suggested to create registries for studies to identify companion diagnostic tests and determine a competitive test for MRD. Consensus is also needed for treatment of multifocal disease; some centers see *EGFR* mutation correlation with multifocal disease, and the data around sublobal resections is unclear.

### Coordinated care

Management of NSCLC requires input from a multidisciplinary team to guide the variety of necessary diagnostic and treatment procedures. Observational studies have shown that coordinated care may accelerate treatment and improve outcomes.^38^ Full integration of cardiothoracic surgeons, thoracic surgeons, radiation oncologists, pulmonologists, pathologists, and oncologists is needed to inform development of comprehensive standards of care. Organization of multidisciplinary patient discussions, tumor boards, and conferences could help delineate appropriate roles across specialties and reduce treatment delays. Additionally, a more active role for surgeons in clinical trials would be beneficial given the large variation in treatment paradigms across institutions, some of which have adopted neoadjuvant as their new standard in contrast to those which primarily employ adjuvant therapy.

Quality measures are needed to increase genetic testing and its associated payer reimbursement. Institutions could form multidisciplinary committees to define and establish metrics so that Centers for Medicare & Medicaid Services (CMS) reimbursement is dependent on genomic testing. The panel suggested creation of a registry that CMS can use to monitor adherence for merit-based reimbursement and shifting the reimbursement paradigm to disease-specific versus specialty-specific metrics. The registry could also be used to measure testing rates.

## Targeting *KRAS/BRAF/MET* and other genomic alterations in NSCLC

Unmet needs identified by the panel are outlined in Table 6

**Table 6.** Key gaps and needs in targeting KRAS/BRAF/MET and other genomic alterations in non-small-cell lung cancer.

1. Genetic screening guidelines and communication between centers, particularly around sequential screening
2. Understand additional driver alterations in NSCLC
3. Better define biomarkers and evaluate co-mutations to guide treatment decisions
4. Develop new drugs or treatment strategies to:
Minimize (physical and financial) toxicity
Overcome resistance (identify resistance mechanisms)
Explore role of immunotherapy
5. New therapies for uncommon alterations (eg, non-G12C KRAS mutations)
6. Identify BRAF V600E therapy of choice
7. Evaluate tolerability of MET inhibitors and optimal predictive biomarkers
8. Study *ALK* and *ROS* subgroup variants; influence on treatment decisions
Establish standard 2L therapy for *ROS + *disease
Chemotherapy/immunotherapy versus chemotherapy alone (+ bevacizumab)
9. Guidelines for *NTRK/RET* fusions testing strategies and communication of results

Abbreviations: ALK, anaplastic lymphoma kinase; NSCLC, non-small cell lung cancer; ROS, receptor tyrosine kinase encoded by the c-ros oncogene.

Targeted therapies are becoming the backbone for patients with uncommon mutations in NSCLC due to improved outcomes, but there are challenges with increased financial costs and developing optimized treatment strategies.^39^ Genomic testing is essential for proper treatment but may be financially inaccessible, and high out-of-pocket costs may be associated with decreased adherence and survival.^40^ Communication between specialties and centers about comprehensive testing can help drive prioritization of RNA versus DNA testing when tissue is limited to avoid sequential screening. Better understanding of driver mutations, definition of biomarkers, and evaluation of co-mutations is needed to inform treatment decisions, particularly when considering immunotherapies, as molecular heterogeneity can impact the biology of disease, and in turn, the therapeutic response.^41,42^ Overall, there is a need for treatment strategies that minimize toxicity, financial burden, and overcome or circumvent mechanisms of resistance.

In the context of specific mutations or fusions, there is a need for new therapies or combinations targeting KRAS beyond G12C. For *BRAF* mutations, there is a need to further explore considerations of *PD-L1* as a biomarker and sequencing of immunotherapy with *BRAF* therapy of choice to improve response rate, citing a recent RW study by Gibson et al.^43^ that revealed how both dual *BRAF/MEK* inhibition and immunotherapy-based regimens have evidence of benefit in *BRAF*-mutated NSCLC. Further, in the context of *BRAF*, it was noted that future studies are needed to evaluate copy number variation and RNA in smoker versus non-smoker populations, and how copy number might affect response to therapy.

With *MET* mutations, questions remain around their impact on treatment (eg, how to bypass resistance, assess tolerability). On a practical level, are there ideal 1L treatments for *MET*-mutated NSCLC? Greater understanding of the cell biology of *MET* amplification, fusions, co-mutations, protein expression, and downstream signaling is needed, and may uncover additional targets of intervention.

For *ALK/receptor tyrosine kinase encoded by the c-ros oncogene* (*ROS1*) mutations, treatment selection and sequencing based on mutation variants and immune landscape could maximize CNS benefit, and optimal sequencing is particularly important with the onset of 4th generation ALK inhibitors (NCT05384626, NCT04849273). Liquid biopsy should be investigated for monitoring to inform sequencing. Additionally, optimal treatments for each of the several recognized variants of *ALK* mutations must be identified. For example, for D1202R mutations, is ivatinimib use recommended, and is anectinib resistance a consideration? Further, there is a knowledge gap around the prognostic value of specific *ROS1* functional variants. For *NTRK/RET* fusions, NGS testing should include both RNA and DNA, and multistage sequencing of increasing panel size may be ideal if a biopsy sample is available.

## Immunotherapy in advanced NSCLC

Key knowledge gaps and controversies are listed in Table 7

**Table 7.** Key gaps and needs in immunotherapy in non-small-cell lung cancer.

1. Implement a multidisciplinary approach to optimize 1L therapies 2. Determine the optimal approach to consolidation therapy 3. Determine the optimal approach for *PD-L1* ≥ 50% and for *PD-L1* < 50% 4. Define the role of ADCs with immunotherapy, considering overlapping toxicities (especially pulmonary) 5. Define acquired immunotherapy resistance in terms of time (early vs late), radiographic signature, biomarkers 6. Should immunotherapy continue through progression, or is it better to rechallenge later?

Questions remain around proper use of immunotherapy both in 1L and as a consolidation therapy, particularly in the context of biomarker (eg, *PD-L1*) expression or combination treatment strategies. Treatment patterns with CTLA-4 agents varied across the panel; examples of use included with adjuvant immunotherapy, among patients with stage 3 disease who progress on durvalumab, and patients with low *PD-L1* who decline chemotherapy. For patients with *PD-L1* ≥ 50%, treatment selection should be based on patient and tumor factors such as age, CNS metastases, and *STK11/KEAP1* mutations. As challenges with toxicities for dual checkpoint blockade compared to dual chemo-immunotherapy exist for these patients, more prospective trials with single agent anti-PD-L1, like the ongoing TRITON study (NCT06008093), are needed. For patients with *PD-L1* < 50%, guidance is needed around appropriate time to add anti-VEGF or anti-CTLA-4 to a chemotherapy/anti-PD-L1 backbone, and what to select for in the case of *PD-L1* < 1%. Additionally, the potential overlapping toxicities of ADCs and immunotherapy must be considered during combination/sequencing strategies, given the demonstrated immunomodulatory effect of ADCs.^44^

A need for standardized definition of early and late acquired resistance versus progression, and how these are best measured, was highlighted. For example, is a time-based, radiographic-based, or molecular-based marker most appropriate? What is the ideal duration off treatment for those who completed immunotherapy? It is also unclear how to assess the role of continued immunotherapy through progression versus rechallenging later. In the case of leptomeningeal disease (LMD), immunotherapy offers promise, given that checkpoint inhibitors may be able to access the CNS and have proven efficacious in RW studies, but these patients are often excluded from pivotal trials.^45^ Increased clinical trial inclusion along with clinical guidance for diagnosis, monitoring, and response assessment, are needed for patients with LMD.

## Discussion

Participants in the *Bridging the Gaps in Lung Cancer* conference explored prevailing challenges, knowledge gaps, and controversies in the field. Across lung cancer types, key themes centered on enhancing biomarker testing, validation, and clinical application; establishing standards of care and larger bodies of evidence around treatment sequencing and combination therapy strategies; and designing trials that can more quickly provide clinically actionable results to inform guidelines and best practice. The need for multidisciplinary collaboration, education, and communication was consistently emphasized.

The *Bridging the Gaps in Lung Cancer* WG will reconvene in 2024 to prioritize identified gaps and formulate consensus recommendations for pressing areas in clinical practice, utilizing a modified Delphi method. The aim is to publish these recommendations as actionable guidance for the lung cancer care community, with the hope of bridging knowledge gaps to enable delivery of optimal care and drive improved outcomes for patients with lung cancer.

## Data Availability

The data underlying this article are available in the article.
